# Siblings With Thrombocytopenia Found To Have a Pathogenic Variant in the NFkB1 Gene

**DOI:** 10.7759/cureus.49168

**Published:** 2023-11-21

**Authors:** Kholoud Bakheet, Saddiq Habiballah, Emtenan Basahl, Ali Algiraigri, Ashwag Alsaidalani, Mohammed Nashawi

**Affiliations:** 1 Pediatrics, King Abdulaziz University Faculty of Medicine, Jeddah, SAU; 2 Pediatric Rheumatology, King Abdulaziz University Hospital, Jeddah, SAU; 3 Pediatric Allergy/Immunology, King Abdulaziz University Hospital, Jeddah, SAU; 4 Immunology Unit, King Fahd Medical Research Center, Jeddah, SAU; 5 Hematology, King Abdulaziz University Faculty of Medicine, Jeddah, SAU; 6 Hematology, King Abdulaziz University Hospital, Jeddah, SAU; 7 Hematology Research Unit, King Fahad Medical Research Center, Jeddah, SAU

**Keywords:** nfkb1, cytopenia, cvid, itp, thrombocytopenia

## Abstract

Immune thrombocytopenic purpura is one of the most common causes of low platelet count in the pediatric population. Secondary thrombocytopenia has a wide differential diagnosis in children, including rheumatological, hematological, and immunological etiologies. Underlying etiologies must be excluded if suspected before labeling the patient as primary thrombocytopenia. Here, we report two siblings with persistent and profound thrombocytopenia. A 10-year-old girl presented with profound and treatment-refractory thrombocytopenia. Given the patient’s family history of thrombocytopenia of unknown pathology in her older brother, immune dysregulation-related thrombocytopenia was suspected. Whole exome sequencing confirmed a previously reported pathogenic variant in the *NFKB1* gene linked to common variable immunodeficiency 12 (CVID-12) diagnosis for both patients.

## Introduction

Immune thrombocytopenic purpura (ITP) is the most common form of primary thrombocytopenia [1]. It is a platelet-destructive disorder. Platelet count is usually below 150,000/ul at the time of diagnosis, with no other systemic involvements. Patients aged two to six years are the most affected. The disease incidence is one in 10,000 children with nearly equal gender distribution [1,2]. A family history of thrombocytopenia raises suspicion toward an inherited cause of thrombocytopenia, as ITP cases are usually sporadic. The formation of autoantibodies against platelet antigens within the reticuloendothelial system leads to the destruction and premature clearance of those platelets. While this explanation is widely acceptable, the exact underlying pathophysiology is still not fully understood and seems to be multifactorial [3]. Depending on the degree of platelet depletion and other risk factors such as trauma, ITP might present with signs of external “mucocutaneous”, or internal “gastrointestinal, urinary, or intracranial” bleeding [3,4]. Despite low platelets, the complete blood count of those patients typically shows normal values of other parameters, including hemoglobin and white blood cells [5]. 

The presence of another cell line abnormality should provoke questioning toward ITP diagnosis [3,5]. Multiple systems could be involved in the pathophysiology of secondary thrombocytopenia in pediatrics. Hemato-oncological, and immune-mediated conditions in addition to infections can lead to thrombocytopenia in this vulnerable population [3]. In addition to ITP, systemic lupus erythematosus (SLE) is an important cause of secondary thrombocytopenia in childhood [6-9].

Another important hidden disease entity to consider in cases of refractory or recurrent ITP is common variable immune deficiency (CVID) disorders [10]. Many CVID patients have an overlap in the clinical phenotypes with other autoimmune diseases as autoimmune disorders were reported in nearly two-thirds of CVID patients regardless of the genetic variants involved [11]. Autoimmune hemolytic anemia (AIHA) and ITP were the most common conditions that have led to the diagnosis of CVID in about half of these cases in a retrospective chart review of over 300 cases [12]. Genetic variants in nuclear factor Kappa-B1 (*NFKB1*) have been linked to common variable immune deficiencies. Recent reports have shown patients with variants of this gene presenting with autoimmunity and lymphoproliferation in high frequency [13].

In this article, we present two siblings with an *NFKB1* variant. First, an adolescent girl with chronic thrombocytopenia was initially diagnosed with ITP but further workup was performed as she lacked response to the conventional management of ITP. Her older brother presented with a similar phenotype, in addition to evidence of lymphoproliferation. Both siblings’ whole exome sequence confirmed the presence of *the NFKB1* variant and were diagnosed with an immune dysregulation disorder.

## Case presentation

Case 1

The first patient (P1) is an 11-year-old girl without a significant past medical history who presented at nine years of age with an abrupt onset of purpura and ecchymosis for two weeks. She had a similar presentation a year earlier, with spontaneous resolution of symptoms. No other symptoms were reported at both times. Her ecchymosis and purpuric lesions were mainly in her arms, legs, and upper back. She was admitted as a suspected case of immune-mediated thrombocytopenic purpura. She initially had no evidence of lymphoproliferation, but she developed mild hepatomegaly and lymphadenopathy later in her disease course.

Initial labs at our hospital showed mild leukopenia, neutropenia, and thrombocytopenia of 2.4 k/ul (normal range 4.5-13.5), 0.2 k/ul (normal range 2-7), and 1 k/ul (normal range 150-450)), respectively (Table 1). Hemoglobin level was normal at 13.4 g/dl (normal range 12.0-15.0). An infectious workup was done to exclude nonimmunological causes of her bi-cytopenia and was completely negative, including tuberculosis, Epstein-Barr virus (EBV), and parvovirus. Severe acute respiratory syndrome coronavirus 2 (SARS‑CoV‑2) testing was also negative. 

**Table 1:** Changes in the lymphocytes and neutrophils of P1 during follow-up

	Day 0	Day 30	Day 60	Day 120	Day 180	Day 270
Automated lymphocytes (normal range 1.5 – 7 K/uL)	1.33	1.56	1.36	1.7	1.92	1.48
Automated Neutrophils Count (normal range 1 – 8 K/uL)	0.39	0.39	0.25	0.59	0.31	0.74

**Figure 1:**
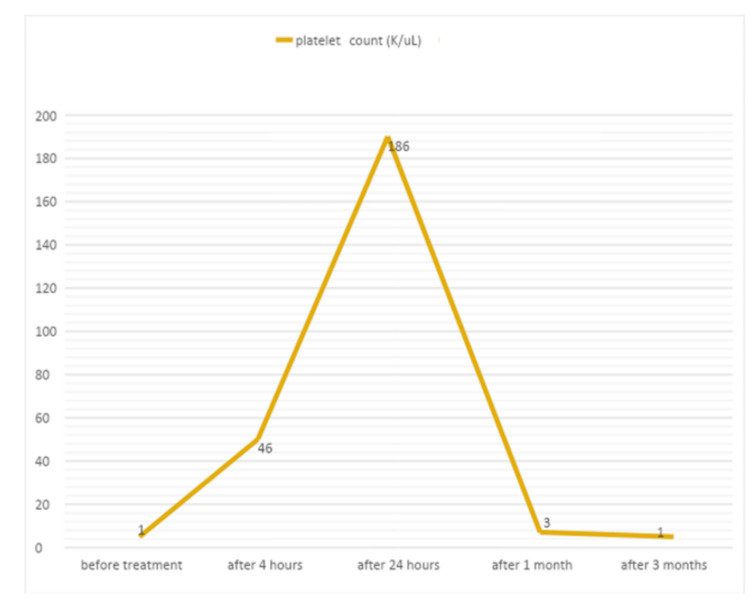
Changes in the platelet count after ITP management for P1 ITP: immune thrombocytopenic purpura

As a case of suspected ITP, she received Rh immunoglobulins of 75 mcg/kg as intravenous immunoglobulin (IVIg) was unavailable at that time. Her platelet counts responded well to that treatment, and she was discharged after 96 hours with a platelet count of 186 k/ul. In less than a month, her platelet count dropped again to 3 k/ul, so she received a second dose of anti-D antibodies, followed by multiple courses of 2 mg/kg/day of oral prednisone (Figure 1). As her platelet counts continued to drop after reducing steroids, she was referred to pediatric rheumatology. 

**Figure 2:**
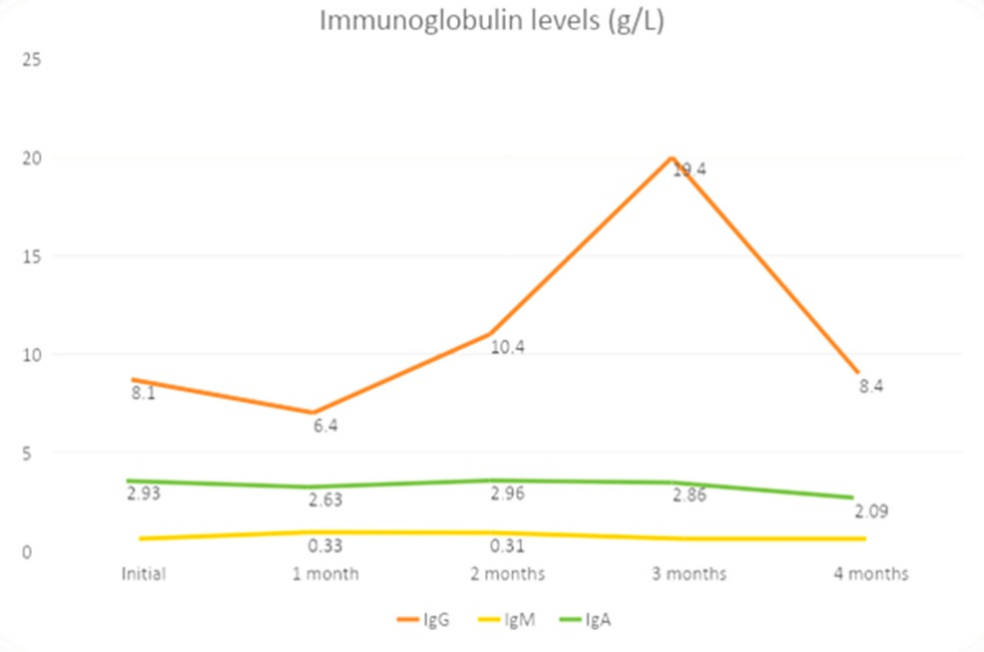
Immunoglobulin levels of first patient during follow-up period of four months

Upon further evaluation, her past medical history was not significant for frequent infections, respiratory symptoms, or complaints, nor did she have skin, mucosal, or joint issues. Her complement levels were normal, the SLE autoantibodies panel was negative, and bone marrow aspiration showed mild hypocellularity with increased megakaryocytes as expected. Expecting immune dysregulation underlying etiology, immune phenotyping was done and showed low IgM 0.33 g/L (normal range 0.5-1.80), CD3 756 μL (normal range 1200-2600), CD4 407 μL (normal range 650-1500), and absence of protective antibodies against tetanus despite her being fully vaccinated for that. Unfortunately, she had received multiple courses of IVIgs at this point, so assessing IgG level was non-informative (Figure 2).

**Figure 3:**
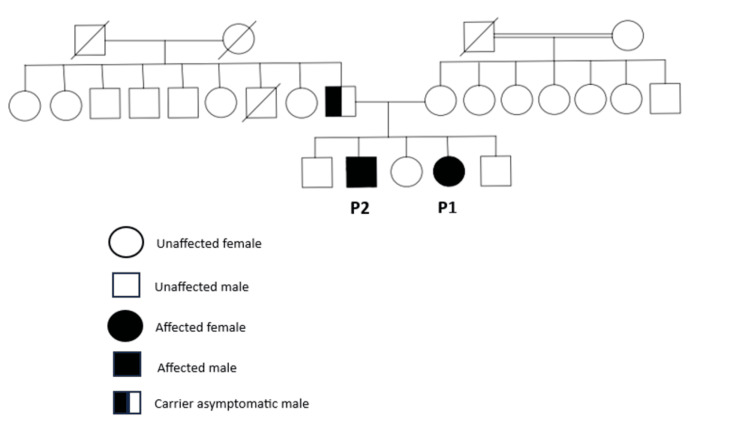
Family pedigree showing carrier and affected individuals

Her family history was interesting in terms of a family member with thrombocytopenia (her brother, referred to as "P2" from now on in this report), in addition to lymphoproliferation (Figure 3).

As the concern for underlying immune dysregulation was highly suspicious, a whole exome sequencing was sent for her, being the kindred here. Her next-generation sequencing revealed a previously reported, disease-causing heterozygous variant in the *NFKB1* gene (c.607C>T, P. Gln203*). 

Case 2

The second patient (P2) is a 21-year-old male, who is a brother of P1 (Figure 3). He was 18 years old when he was found to have thrombocytopenia and hepatosplenomegaly incidentally as he was completely asymptomatic. As tuberculosis is endemic in the area, and in the absence of any evidence of another underlying etiology, he was started on an anti-tuberculosis regimen. While on that regimen, he developed mild and transient transaminitis that resolved with no sequala but persisted to be thrombocytopenic, lymphopenic, and neutropenic, in addition to his generalized lymphadenopathy and hepatosplenomegaly. Despite those findings, he continues to be asymptomatic and free of infections, so no specific treatment was started. Limited immune phenotyping was done and revealed an IgG level of 495 mg/dl (540-1822), with protective antibodies against mumps, rubella, and hepatitis B. After the presence of *the NFKB1* gene variant was confirmed in his sister, the family segregation study uncovered the same variant for him, a heterozygous variant of *the NFKB1* gene (c.607C>T, P.Gln203*). The father’s testing was also positive for the same variant, but he was completely asymptomatic, and his complete blood count result was normal. 

As the genetic variant was confirmed for P2, and as he continued to show evidence of autoimmunity and lymphoproliferation, further laboratory and radiological evaluations were carried out. Immune phenotyping showed an IgG level of 486 mg/dl (normal range, 540-1822 mg/dl), normal IgM and IgA levels, and protective titers against measles, mumps, and tetanus vaccines. His lymphocyte subset showed severe CD4+ T-cells lymphopenia at 277 cells/uL (normal range, 430-1800 cells/uL), and low CD16+ NK-cells at 21 cells/uL (normal range, 78-470 cells/uL). CD8+ T-cells and CD19+ B-cells were normal in the setting of an absolute lymphocyte count of 900 cells/uL (normal range, 900-3100 cells/uL), but unfortunately B cell memory panel was not available. His absolute neutrophil count was low at 0.59 X 10^9^/L (normal range, 1.7-7.0), and his platelet count was 41 K/UL (normal range, 150-450 K/UL). 

A high-resolution chest computed tomography scan for both siblings showed normal results. Both patients started on Ig replacement therapy in addition to azathioprine and are under active treatment and follow-up currently. P1 did not show optimal response to azathioprine, so she was switched to rituximab, while P2 continued on azathioprine pending further follow-up.

## Discussion

CVID disorders are a group of diseases with primary B cell defects, and multisystem involvement [14]. The usual age of presentation for CVID is around adolescence and early adulthood years. Males and females are equally affected. It is sporadic in most cases. A wide range of clinical manifestations have been identified including recurrent infections involving the sinopulmonary or gastrointestinal system. Deep-seated infections, allergic diseases, improper response to antibiotics or vaccines, and autoimmune diseases were also reported [12,15]. As immune dys-regulatory disorders, patients with those diseases can present with granulomatous inflammation of the lung, lymph nodes, and solid tumors such as lymphomas due to their lack of host immunity [15].

Genetic testing should be highly considered in patients presenting with immune deficiency/dys-regulatory concerns. To diagnose a patient with CVID, the European Society for Immunodeficiencies (ESID) proposed diagnostic criteria that required a low IgG level, either low IgA or IgM, in addition to evidence of specific antibody deficiency [14]. Over the past few years, more monogenetic causes of CVID have been described, but the majority of cases still with no identifiable genetic defect. *NFKB1* is one of the identified genes harboring disease-causing variants. This disease is classified as a CVID-12 phenotype. In a recent cohort, 105 heterozygous variants were identified causing variable clinical presentations. The presentation ranged from infectious susceptibility, autoimmunity, and immune dysregulation [13]. As P1 already received multiple courses of IVIg and anti-CD20 treatment, in addition to no pre-treatment immunophenotyping, we could not assess for the ESID proposed diagnostic criteria for CVID. Instead, she was diagnosed with CVID-12 based on her clinical presentation with autoimmunity and lymphoproliferation evidence, in addition to her identified genetic variant. Her brother was also labeled as a CVID-12 patient given his clinical presentation, and the genetic variants he harbors, but his Igs were low prior to treatment. 

The identified variant in our patients (*NFKB1*, P.Gln 203*) has been reported as pathogenic in one female patient who presented with CVID/autoimmune lymphoproliferative syndrome (ALPS) phenotype [13]. Autoimmunity is reported in nearly two-thirds of CVID patients, with almost half of them presenting with autoimmune cytopenia [11,13]. Other common immune-mediated diseases associated with CVID are rheumatoid arthritis, psoriasis, celiac and thyroiditis [16]. Joint manifestations resembling rheumatoid arthritis or juvenile idiopathic arthritis occur in 1-10 % of patients with CVID [16].

SLE with CVID is uncommon, but patients may develop SLE features as a complication of immune dysregulation of CVID [17]. Hypogammaglobulinemia may delay the diagnosis of autoimmunity in CVID patients as auto-antibodies (including antinuclear antibody (ANA) and rheumatoid factors) may be impeded [18]. The mainstay of CVID-12 (NFKB1 deficiency) treatment is Ig replacement and immune suppressive therapy as needed per individual patients [13]. Hematopoietic stem cell transplantation is an option that still does not have enough data in patients with CVID-12 [13]. It is also important to closely follow patients with evidence of immune dysregulation as they are at higher risk of developing malignancies such as lymphomas.

## Conclusions

By presenting these two cases, we are reinforcing the importance of re-questioning the diagnosis of chronic and replacing thrombocytopenia in the presence of other evidence such as lymphoproliferation and family history in our scenario. Moving to genetic testing is strongly recommended in such cases to unveil the underlying pathology. 

## References

[1] Terrell DR, Beebe LA, Vesely SK, Neas BR, Segal JB, George JN (2010). The incidence of immune thrombocytopenic purpura in children and adults: a critical review of published reports. Am J Hematol.

[2] D'Orazio JA, Neely J, Farhoudi N (2013). ITP in children: pathophysiology and current treatment approaches. J Pediatr Hematol Oncol.

[3] Arnold DM (2015). Bleeding complications in immune thrombocytopenia. Hematology Am Soc Hematol Educ Program.

[4] Park YH, Kim DY, Kim S (2022). Management of immune thrombocytopenia: 2022 update of Korean experts recommendations. Blood Res.

[5] Cines DB, Blanchette VS (2002). Immune thrombocytopenic purpura. N Engl J Med.

[6] Schmugge MR, Hiraki L, Rand ML, Blanchette VS, Silverman ED (2003). Thrombocytopenia and thromboembolism in pediatric systemic lupus erythematosus. J Pediatr.

[7] Michel M, Lee K, Piette JC, Fromont P, Schaeffer A, Bierling P, Godeau B (2002). Platelet autoantibodies and lupus-associated thrombocytopenia. Br J Haematol.

[8] Kamphuis S, Silverman ED (2010). Prevalence and burden of pediatric-onset systemic lupus erythematosus. Nat Rev Rheumatol.

[9] Hepburn AL, Narat S, Mason JC (2010). The management of peripheral blood cytopenias in systemic lupus erythematosus. Rheumatology (Oxford).

[10] Park MA, Hagan JB, Maddox DE, Abraham RS (2008). Common variable immunodeficiency: a new look at an old disease. Lancet.

[11] Wang J, Cunningham-Rundles C (2005). Treatment and outcome of autoimmune hematologic disease in common variable immunodeficiency (CVID). J Autoimmun.

[12] Mormile I, Punziano A, Riolo CA Common variable immunodeficiency and autoimmune diseases: a retrospective study of 95 adult patients in a single tertiary care center. Front Immunol.

[13] Lorenzini T, Fliegauf M, Klammer N (2020). Characterization of the clinical and immunologic phenotype and management of 157 individuals with 56 distinct heterozygous NFKB1 mutations. J Allergy Clin Immunol.

[14] Resnick ES, Moshier EL, Godbold JH, Cunningham-Rundles C (2012). Morbidity and mortality in common variable immune deficiency over 4 decades. Blood.

[15] Chapel H, Lucas M, Lee M (2008). Common variable immunodeficiency disorders: division into distinct clinical phenotypes. Blood.

[16] Bloom KA, Chung D, Cunningham-Rundles C (2008). Osteoarticular infectious complications in patients with primary immunodeficiencies. Curr Opin Rheumatol.

[17] Fernández-Castro M, Mellor-Pita S, Citores MJ (2007). Common variable immunodeficiency in systemic lupus erythematosus. Semin Arthritis Rheum.

[18] Wehr C, Gennery AR, Lindemans C (2015). Multicenter experience in hematopoietic stem cell transplantation for serious complications of common variable immunodeficiency. J Allergy Clin Immunol.

